# Ultra-Rapid Crystallization of L-alanine Using Monomode Microwaves, Indium Tin Oxide and Metal-Assisted and Microwave-Accelerated Evaporative Crystallization

**DOI:** 10.5101/nbe.v9i2.p112-123

**Published:** 2017-05-27

**Authors:** Carisse Lansiquot, Zainab Boone-Kukoyi, Raquel Shortt, Nishone Thompson, Hillary Ajifa, Bridgit Kioko, Edward Ned Constance, Travis Clement, Birol Ozturk, Kadir Aslan

**Affiliations:** 1 Department of Chemistry, Morgan State University, 1700 East Cold Spring Lane, Baltimore, MD 21209, USA; 2 Department of Physics and Engineering Physics, Morgan State University, 1700 East Cold Spring Lane, Baltimore, MD 21209, USA

**Keywords:** ITO, Evaporative crystallization, Silver island films, Microwave heating

## Abstract

The use of indium tin oxide (ITO) and focused monomode microwave heating for the ultra-rapid crystallization of L-alanine (a model amino acid) is reported. Commercially available ITO dots (< 5 mm) attached to blank poly(methyl)methacrylate (PMMA, 5 cm in diameter with 21-well silicon isolators: referred to as the iCrystal plates) were found to withstand prolonged microwave heating during crystallization experiments. Crystallization of L-alanine was performed at room temperature (a control experiment), with the use of two microwave sources: a 2.45 GHz conventional microwave (900 W, power level 1, a control experiment) and 8 GHz (20 W) solid state, monomode microwave source with an applicator tip that focuses the microwave field to a 5-mm cavity. Initial appearance of L-alanine crystals and on iCrystal plates with ITO dots took 47 ± 2.9 min, 12 ± 7.6 min and 1.5 ± 0.5 min at room temperature, using a conventional microwave and focused monomode microwave heating, respectively. Complete evaporation of the solvent using the focused microwaves was achieved in 3.2 ± 0.5 min, which is ~52-fold and ~172-fold faster than that observed at room temperature and using conventional microwave heating, respectively. The size and number of L-alanine crystals was dependent on the type of the 21-well iCrystal plates and the microwave heating method: 33 crystals of 585 ± 137 μm in size at room temperature > 37 crystals of 542 ± 100 μm in size with conventional microwave heating > 331 crystals of 311 ± 190 μm in size with focused monomode microwave. FTIR, optical microscopy and powder X-ray diffraction analysis showed that the chemical composition and crystallinity of the L-alanine crystals did not change when exposed to microwave heating and ITO surfaces. In addition, theoretical simulations for the binding of L-alanine molecules to ITO and other metals showed the predicted nature of hydrogen bonds formed between L-alanine and these surfaces.

## Introduction

Crystallization is an important technique consistently utilized within the food, chemical and pharmaceutical and biotechnology industries [1, 2]. In order to develop new products, and/or improve or purify existing products, researchers worldwide continue to develop quicker and more efficient ways to produce crystals [3]. Evaporative crystallization, in which nucleation and crystal growth occurs when the solution evaporates, is a widely used chemical solid–liquid separation technique [4]. Evaporative crystallization is typically employed at small-scale processes where solvent can be removed in a cost-effective manner to produce highly-desirable crystals. However, a drawback of evaporative crystallization is the requirement of highly soluble compounds to grow high quality of crystals in large quantities [5]. One can find numerous published reports on the applications of evaporative crystallization, such as, carbon nanotubes [6], polymers [7], amino acids [8, 9, 10], salts [11], and other important chemical compounds [12, 13, 14]. Despite the advantages afforded by the evaporative crystallization technique, such as solvent evaporation by heating and relatively simple instrumentation, there is still a need for improvements for the growth of crystals of desired products in a speedy, repeatable and high-throughput fash ion.

In response to the need for better crystallization techniques as describe above, The Aslan Research Group introduced and demonstrated a novel crystallization technique, called Metal-Assisted and Microwave-Accelerated Evaporative Crystallization (MA-MAEC) [8]. The crux of the MA-MAEC, which was previously described in detail elsewhere [8, 15, 16], is based on the use of low power microwaves and metal nanoparticle and thin film surfaces to accelerate the rate of crystallization. In this regard, metals and metal oxides, such as, gold, silver, nickel, copper, ITO is deposited on to a planar blank glass or polymer (PMMA) or polyethylene terephthalate (PET). Metal nanoparticles on a planar surface provide selective nucleation sites and a microwave-transparent medium for the thermal gradient between the warmer solution and the metal surface that allows for crystallization to occur [8]. The MA-MAEC technique was demonstrated to significantly increase the size of the target crystals while significantly decreasing the crystallization time. The reader is referred to the references provided herein for additional information regarding the MA-MAEC technique [8, 15, 16].

In one of our recent publications [17], we demonstrated that iCrystal plates with ITO are the most efficient surfaces for the growth of biological molecules in terms of control over crystal size, number and quality using a 100 W monomode microwave source at 2.45 GHz and the MA-MAEC technique to date. ITO has a low resistance and great optical transmittance within the visible section of the electromagnetic spectrum, consequently giving rise to many different uses on the market today, such as, optical coatings on for light emitting devices [18] and biological binding events [19, 20]. In addition, the preparation of ITO films is carried out by well-established industrial techniques: insignificant variation in the nanoparticle density on the surface are achieved in a consistent manner, which makes ITO films very attractive surfaces for use in microwave-accelerated processes. On the other hand, when exposed to microwaves, electric charge can accumulate on metal and metal oxide surfaces that are in similar size to the wavelength of the microwave source (for example, the wavelength of microwaves 2.45 GHz and 8 GHz is ~12.2 cm and ~3.75 cm, respectively). In this regard, the size of the metal thin films or the surface area for the metal nanoparticles are deposited to be used with the MA-MAEC technique was reduced to 1/10^th^ of the wavelength of the microwave source. In addition, we also demonstrated that the type of microwave source can influence the crystallization time, the size and the quality of crystals grown on iCrystal plates. To date, two types of microwave sources were used by our research group for the crystallization studies using the MA-MAEC technique: 1) a multi-mode microwave source operating at 2.45 GHz (in a conventional microwave oven, fixed power 700 W-1200 W) [21] and 2) solid-state monomode microwave source operating at 2.45 GHz (in iCrystal system, variable power up to 100 W) [17]. The studies discussed above revealed that the use of a monomode microwave source at 2.45 GHz of 100 W microwave power resulted in the reduction of crystallization time, despite the fact that the microwave power is significantly lower than that of a conventional microwave oven, which can be attributed to the homogeneous heating of the samples by monomode microwave heating. We note that even though the applied power in conventional microwave oven is higher, the power density is typically lower than those in monomode microwave systems due to the differences in the size of the microwave cavities. These observations motivated us to further investigate the crystallization of biological materials with the use of monomode microwave heating at a different microwave frequency and ITO surfaces.

In this paper, we report the ultra-rapid crystallization of L-alanine (a model amino acid) using ITO thin films and focused monomode microwave heating at 8 GHz and 20 W. We note the difference of this study from our previous publications is the use of focused microwave heating: the applicator tip of the microwave source used affords for the focusing of microwaves to a small cavity of 5 mm (diameter) × 2 mm (depth) in size. ITO dots of 5 mm dimeter (produced from the commercially available large size ITO thin films) were attached to each well of the iCrystal plates (also 5 mm in diameter) based on our observations that larger sized ITO (>50 mm) deteriorates quickly when exposed to microwaves. The initial and final time for the crystallization of L-alanine on iCrystal plates with ITO dots and blank iCrystal plates (no ITO, a control surface) at room temperature, using a conventional microwave at power level 1 and using a 20 W focused monomode microwave source were recorded and compared. FTIR, optical microscopy and powder X-ray diffraction analysis were used to assess and ensure the chemical composition of the L-alanine did not change when exposed to microwave heating and ITO. Theoretical simulations of the binding of L-alanine to ITO and other common metal and metal oxides were carried out to predict the binding energy and nature of hydrogen bonds formed between L-alanine and metal and metal oxide surfaces.

## Materials and Methods

### Materials and instrumentation

L-alanine was purchased from Sigma-Aldrich; WI, USA. Silicon isolators with 21-wells capacity (5 cm in diameter and 2 mm in thickness, diameter of each well in 5 mm) were designed by the Aslan Research Group and manufactured by Grace BioLabs (Oregon, USA). ITO-coated PET films (Item number: ITO-PF-14K-300300, PET film: 300 mm width × 1000 mm length × 0.175 mm thickness were obtained from MTI Corporation (California, USA). The thickness of ITO layer is 115 nm ± 10 nm, resistivity of ITO is 14 ohm/sq, transparency is > 75% and haze is 3%). Blank PMMA disks (5 cm in diameter) were purchased from McMaster-Carr (Illinois, USA).

Deionized (DI) water was purified using Millipore Direct-Q UV apparatus. Emerson microwave (fixed power of 900 Watts, Model no. 9339SB) was purchased from Walmart, Inc. AR, USA. Monomode microwave with applicator tip variable power up to 20 W, 8 GHz (Model ISYS800) was purchased from Emblation Microwave (Scotland, UK). ITO films on iCrystal plates were analyzed using a Phenom XL scanning electron microscope (SEM) that was purchased from Nanoscience Instruments (Alexandria, VA, USA). Optical microscope (Swift, 5 MP) was purchased from Microscope World, Carlsbad, CA, USA).

## Methods

### Preparation of iCrystal plates with and without ITO surfaces

**Fig. 1:**
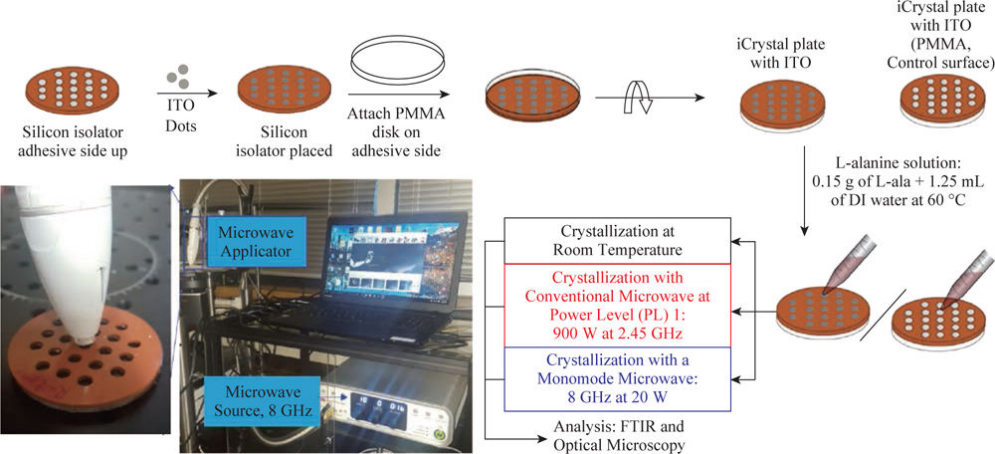
Schematic depiction of experimental setup for the crystallization of L-alanine on iCrystal plates with and without ITO using the MA-MAEC technique at room temperature (a control experiment), using conventional microwave heating (900 W, power level 1, a control experiment) and using a monomode microwave source (8 GHz at 20 W). ITO film is deposited onto polyethylene terephthalate (PET) was cut into smaller pieces. A photograph of our experimental setup with the 8 GHz monomode microwave source is shown.

ITO films were cut out into ~2 mm dots using a hole puncher and placed on to adhesive side of the silicon isolators to cover each well individually, that is, wells are filled with ITO dots and were placed on the iCrystal plates (Fig. 1). Subsequently, blank PMMA disk was also placed on the adhesive side of the silicon isolators to create an air-tight and water-tight seal. The size of the ITO dots (2 mm) eliminated the damage occurred during microwave heating. iCrystal plates without ITO dots were used as control surfaces to assess the effect of using ITO in the MA-MAEC technique.

### Preparation of L-alanine solution

Fresh L-alanine solution was prepared by completely dissolving 0.15 g of L-alanine in 1.25 mL of deionized water at 60 °C before use. Solubility of L-alanine in water is 0.165 g/mL and 0.218 g/mL at room temperature and at 50 °C, respectively.

### Crystallization of L-alanine

A 30 μL portion of the freshly prepared L-alanine solution at 60 °C was pipetted into each well on the iCrystal plates. Each well was then viewed under a microscope to ensure that there were no initial crystals in the solution. In the crystallization experiments at room temperature, an initial picture of one well was taken, and then successive pictures were taken every 5 minutes by using auto-capture until complete evaporation of the solvent was achieved. Pictures of the remaining 20 wells were taken once crystallization was complete. In the crystallization experiments using the conventional microwave, the iCrystal plates were placed in the previously marked position in the center of the microwave oven for 5 minutes at power level 1. A picture of that same well was taken every 5 min until complete evaporation of the solvent was achieved. A picture of the remaining 20 wells was taken when crystallization was completed. In the crystallization experiments using monomode microwave (8 GHz monomode microwave), microwave heating of L-alanine solution was carried out at 20 W. One well containing the L-alanine solutions was microwaved at 30 sec intervals and then a picture was taken, and microwave heating of the well was continued until the complete evaporation of solvent was achieved. The process was repeated for the remaining 20 wells and final pictures of all wells were taken. Each experiment in this study was repeated at least three times and the average values with standard deviations were reported.

### FTIR analysis of L-alanine crystals and platforms

L-alanine crystals grown on iCrystal plates were analyzed using Fourier transform infrared (FTIR) spectroscopy. The ITO on iCrystal plates were analyzed by using an SEM after experiments were completed to assess the damage on the surface.

### Powder X-ray diffraction (XRD) and Image-J analysis of L-alanine crystals

Powder XRD data was collected with Rigaku MiniFlex. The total surface area and crystal count for each optical image of L-alanine crystals were quantified and measured with the use of the Image-J software (a free image processing and analysis software in Java). The crystal length and total surface area for all optical images of L-alanine crystals were calculated using the Image-J software.

### Theoretical simulations

L-alanine is known to crystallize in an orthorhombic lattice with space group P_212121_. There are four L-alanine molecules in the unit cell linked by a three-dimensional network of hydrogen bonds. The unit cell dimensions (a = 6.032, b = 12.343, c = 5.784; α = β = γ = 90°) were obtained from the Cambridge Structural Database (CSD) and the calculations were carried out using Biovia Materials Studio 8. The details of these calculations were provided in a previous publication [22].

## Results and Discussion

### Theoretical simulations

**Fig. 2:**
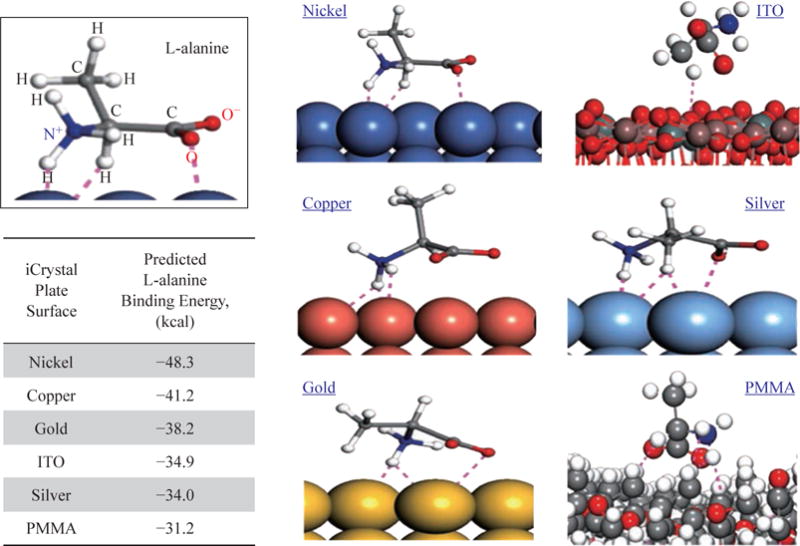
Simulation of L-alanine molecule binding with various materials performed and the calculated binding energy using Materials Studio software. Dashed lines indicate close contacts (i.e., summation of all forces: London forces, dispersion forces and Van der Waals forces) between L-alanine molecules and metal surfaces.

Fig. 2 depicts the simulation of binding of L-alanine on different surfaces after a stable bond was predicted to form and the calculated binding energies using Materials Studio software. Our calculations predict that the largest binding energy for L-alanine on the materials investigated in this study is required for PMMA (−31.2 kcal). In addition, binding energy for L-alanine on silver and ITO was predicted to be similar, −34.0 kcal and −34.9 kcal, respectively. Significant decrease in binding energy was predicted for gold, copper and nickel surfaces, where the lowest binding energy (−48.3 kcal) was calculated for nickel surfaces. These predictions were further investigated by studying the orientation of L-alanine molecule on the surfaces used. Two charged groups (–NH_3_^+^ and –COO^−^) and the central –CH group of L-alanine are predicted to form hydrogen bonds with the nickel surface and the hydrophobic –CH_3_ group is oriented away from the nickel surface. While the similar hydrogen bonds are predicted form between the charged groups and copper, gold and silver, the hydrophobic –CH_3_ group moves closer to the metal surface (–CH_3_ group is furthest away from copper and is nearest to silver surface). Subsequently, additional hydrogen bonds between the –CH_3_ group of L-alanine and silver surface are predicted to occur, which can explain the predicted increase in the binding energy for L-alanine on silver surfaces. Our simulations predict no hydrogen bonds between L-alanine and ITO and PMMA surfaces. The hydrophobic –CH_3_ group of L-alanine is predicted to be oriented away from the PMMA surface, while the orientation of L-alanine on ITO is like that is predicted for silver without the hydrogen bonds, as evidenced by the similar binding energies for ITO and silver. These predictions provide insights to molecular binding events on different surfaces, which is critically important in the design of surfaces for use with the MA-MAEC technique. In addition, preparation of surfaces with ease, homogeneous nanoparticle distribution, repeatability of preparation and large surface area samples are also important factors to be considered for crystallization of molecules of interest. In this regard, ITO films, which are developed for solar energy technology, are proven successful in all the criteria mentioned above. The Aslan Research Group have previously demonstrated the use of silver, gold, nickel and copper for rapid crystallization of amino acids in a recent publication [23].

### Evaluation of ITO for use with MA-MAEC technique

Based on the proven advantages afforded by ITO, the focus of the current work is demonstration of the proof-of-principle use of ITO for ultra-rapid crystallization of L-alanine (a model amino acid) using the MAMAEC technique. To appropriately use commercially available ITO films (in 30 cm wide) with the iCrystal plates (5 cm diameter) for the rapid crystallization of L-alanine using the MAMAEC technique, it is important to determine the optimum size of the ITO films that can withstand microwave heating. In this regard, we initially tested whether 5 cm circular ITO films affixed to the iCrystal plates can withstand microwave heating. Fig. S1 (Supplementary Information) shows our initial testing of ITO films under different power levels and times using a 2.45 GHz conventional microwave. ITO films (~4 cm) underwent significant physical damage when exposed to microwave heating within 30 seconds at power level 1 and power level 10. These observations can be attributed to similarity of the wavelength of microwaves at 2.45 GHz (~12.2 cm) to the size (~4 cm) of circular ITO films, where charge buildup in the ITO films exposed to heterogeneous microwave field occurs (Fig. S2(a)).

**Fig. 3:**
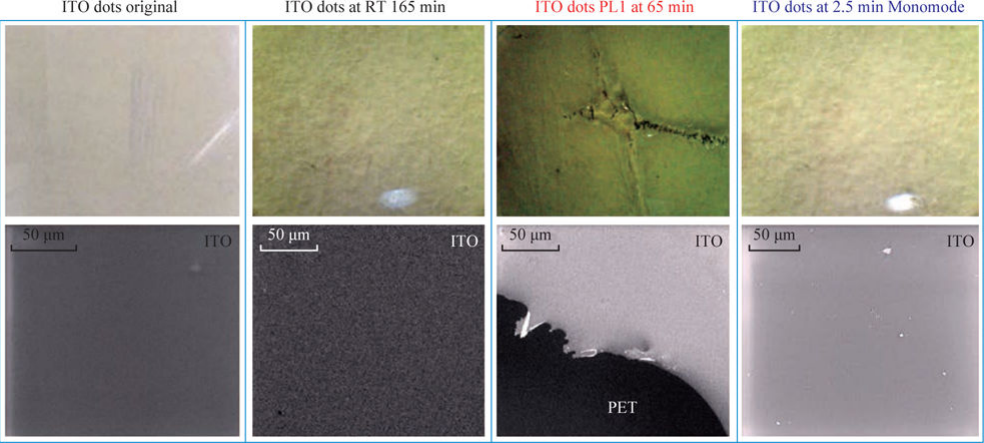
Optical and SEM images of ITO on iCrystal plates before (ITO original), at room temperature (RT) after 165 min and after exposure to conventional microwave heating at 65 min (PL 1) and a monomode microwave heating at 2.5 min. ITO film is on polyethylene terephthalate (PET). All surfaces were dry.

It was also previously shown that metal thin films can withstand microwave heating without suffering significant damage when their size is less than 1/10^th^ of the wavelength of the microwaves [24, 25]. In this regard, we reduced the size of the ITO films to 5 mm (i.e., ITO dots) and investigated whether ITO dots can withstand continuous microwave heating. Fig. 3 shows the optical microscope and SEM images of ITO dots exposed to different experimental conditions. ITO dots before exposure to microwaves used as a control surface for the comparison and assessment of damage on ITO dots after exposure to microwaves. ITO dots kept at room temperature for 165 min show minor granulation at the surface. Fig. 3 shows that significant damage occurred to the ITO dots after microwave heating for 65 min damage at power level 1 in a conventional microwave, where ITO was removed from the surface exposing the PET under-layer. Fig. 3 also shows that the exposure of ITO dots to monomode microwave heating at 8 GHz for 2.5 min did not result in significant damage to the surface. It is important to note that the microwave heating times used in Fig. 3 correspond to the complete evaporation times observed during crystallization experiments, which indicates whether the physical stability of ITO films are sufficient for use with the MA-MAEC technique.

### Crystallization of L-alanine

**Fig. 4:**
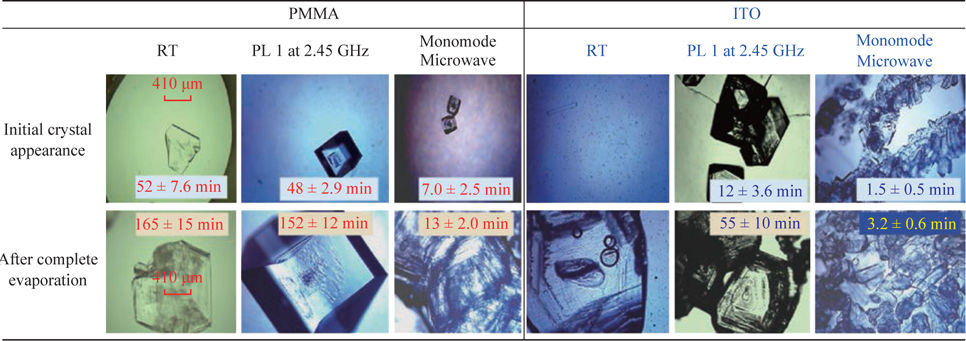
Optical images of L-alanine crystals after their initial observable appearance and complete evaporation of the solvent on iCrystal plates with and without ITO (i.e., PMMA) at room temperature (RT) and after exposure to conventional microwave heating (Power Level, PL 1) and a monomode microwave heating. Scale bar = 410 mm and applies all optical microscopy images in this figure.

Fig. 4 shows the comparison for the average time for initial crystal appearance (i.e., the size of the crystal reached ~50 mm) and the average time for complete evaporation of solvent on the two versions of the iCrystal plates (blank PMMA and ITO) surfaces under different experimental conditions. At room temperature, initial L-alanine crystals of measurable size with the optical microscope used appeared on the PMMA and ITO surfaces at 52 ± 7.6 min and 47 ± 2.9 min, respectively. Complete evaporation of solvent (where solvent is visually absent from the surface) at room temperature occurred on PMMA and ITO after 165 ± 15 min and 160 ± 8.7 min, respectively. In addition, the use of conventional microwave heating at power level 1 resulted in the complete evaporation time on PMMA surfaces, as previously reported (we note that these results are provided for the sake of comparison). The use of ITO on the iCrystal plates and conventional microwave heating also resulted in a 3-fold decrease in the complete evaporation time (from 160 ± 8.7 min and 55 ± 10 min) as compared to the identical experiments carried out at room temperature. We note that all experiments in this study were repeated a minimum of three times to demonstrate the repeatability of the data. In addition, the optical images of L-alanine crystals grown on the three different iCrystal plates with ITO dots and focused monomode microwave heating demonstrate the repeatability of the MA-MAEC technique (Fig. S3).

It is also important to comment on the differences between the microwave sources used in this study. Microwaves with lower frequencies have can penetrate deeper in to the samples than the microwaves with higher frequencies, described as in the following statements [26, 27]: microwaves at 915 MHz and 2.45 GHz are suited for large volume heating (for example, 1,000 mL beaker in a conventional microwave oven with a 30 cm cavity) [28], while the use of higher frequencies is typically used for small volume heating. In this regard, higher frequency microwave heating in the range 5.8 GHz-10 GHz is ideal for near surface based heating, such as, heating of the samples in the iCrystal plates (2 mm depth). Conventional microwave has a large size cavity and a rotating plate to alleviate the effect of heterogeneous distribution of electromagnetic energy from the microwave source (i.e., multi-mode klystron technology): in this configuration, the iCrystal plates are heated in a relatively homogeneous manner. To study the effect of use of focused microwaves from a monomode solid state source, where microwave energy is focused to a small cylindrical area (5 mm in diameter, 2 mm for iCrystal plates), a commercially available monomode microwave source (8 GHz, up to 20 W) equipped with an applicator tip was employed with the MA-MAEC technique. We note that the choice of frequency and power is based on current availability of suitably priced solid-state microwave technology.

Fig. 4 shows that the use of focused monomode microwaves and the iCrystal plates with ITO results in the appearance of L-alanine crystals as early as in 1.5 ± 0.5 min and a complete evaporation of solvent in 3.2 ± 0.6 minutes, which represents a ~52-fold and ~17-fold decrease as compared to complete evaporation of solvent on blank iCrystal plates (PMMA) at room temperature and using monomode microwave heating, respectively. Fig. 4 also show that the size of the L-alanine crystals grown at room temperature and using conventional microwave heating are significantly larger than those grown using a monomode microwave source. These observations can mainly be attributed to the differences in the rate of evaporation of solvent: slowest at room temperature, faster using conventional microwave heating and fastest using focused monomode microwave heating. We also note that the slight decrease in the crystallization time on ITO at room temperature can be directly attributed to the presence of ITO or metal-assisted evaporative crystallization. Since the initial temperature of the L-alanine solution is 60 °C and the solution is rapidly cooled as soon as the experiments is commenced at room temperature, ITO functions mainly as selective nucleation sites. The real impact of ITO is realized when used in combination with microwave heating, where a consistent microwave-induced temperature gradients exist between the solvent and ITO surface.

**Fig. 5:**
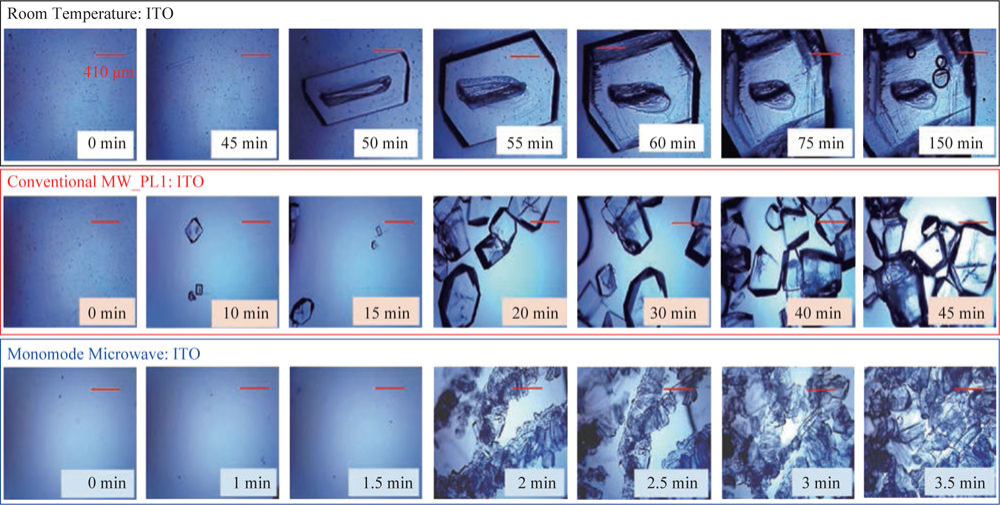
Timed optical images of L-alanine crystals grown on iCrystal plates with ITO at room temperature and after exposure to conventional microwave heating (Power Level, PL 1) and a monomode microwave heating.

The growth of L-alanine on ITO was further investigated by monitoring the size of L-alanine crystals during the evaporation of solvent at room temperature, conventional microwave heating and monomode microwave heating, as shown in Fig. 5. At room temperature, L-alanine crystals first appeared at ~45 min and grew rapidly in size until the complete evaporation of solvent in ~150 min. In a conventional microwave at power level 1, crystallization of L-alanine began as early as ~10 min and ended at ~45 min after the complete evaporation of solvent. When monomode microwave heating was employed, numerous L-alanine crystals appeared within ~2 min, which represents up to 10-fold decrease in the time of appearance and growth of L-alanine crystals as compared to conventional microwave heating. Moreover, the number of L-alanine crystals continued to grow until the solvent was completely evaporated in ~3.5 min. These observations imply that one can grow L-alanine crystals of desired size by intermittent or continuous in situ imaging of the iCrystal plates during the crystallization process. That is, one can stop the crystallization experiments by removing the solvent when the desired size of crystals is achieved.

### Reproducibility studies

**Fig. 6:**
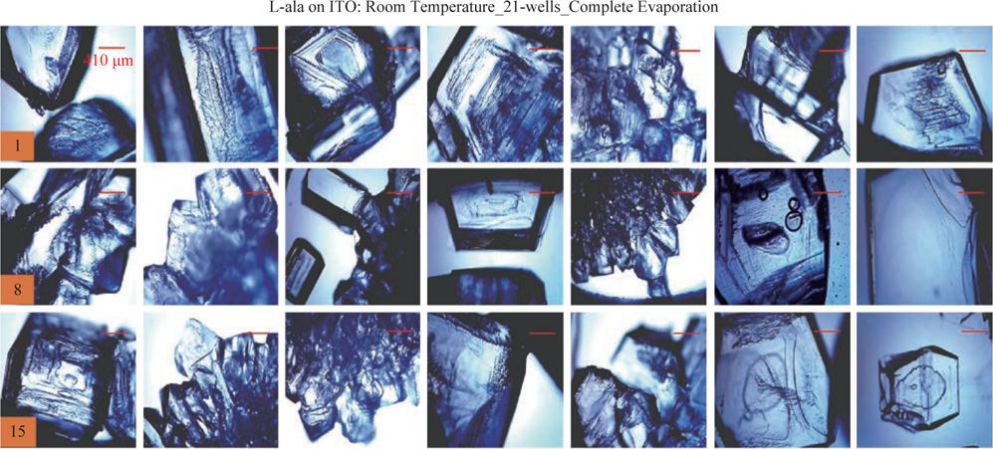
Optical images of L-alanine crystals grown on the 21 wells of the iCrystal plates with ITO at room temperature after the complete evaporation of the solvent. Numbers in the orange boxes indicate the number of wells in the iCrystal plates.

**Fig. 7:**
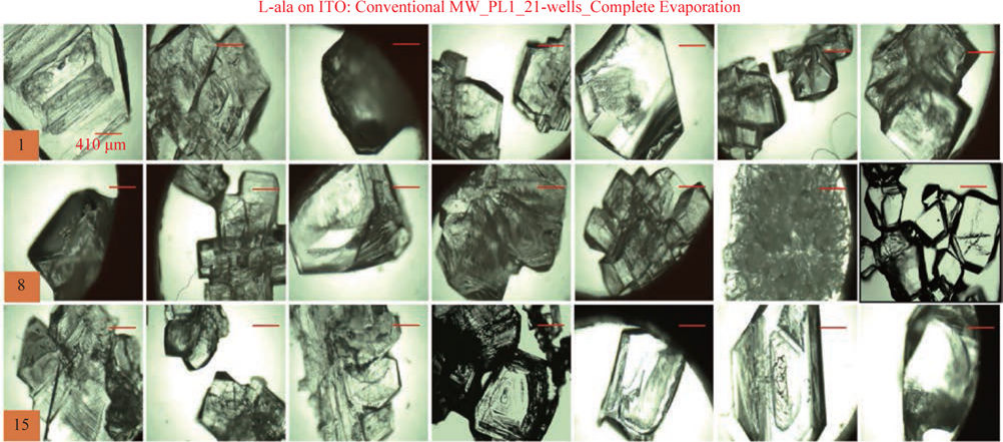
Optical images of L-alanine crystals grown on the 21 wells of the iCrystal plates with ITO using conventional microwave heating after the complete evaporation of the solvent.

**Fig. 8:**
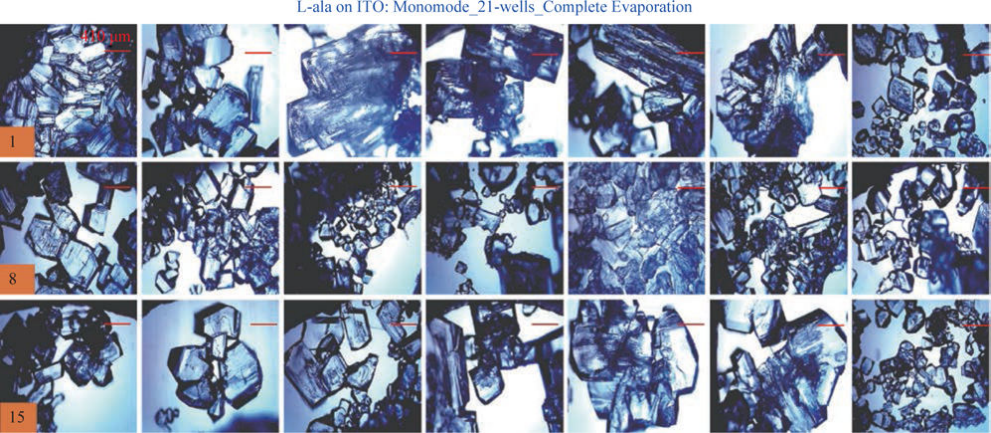
Optical images of L-alanine crystals grown on the 21 wells of the iCrystal plates with ITO using monomode microwave heating after the complete evaporation of the solvent.

For the MA-MAEC technique to be considered as an efficient and acceptable crystallization technique by scientists worldwide, it is critical to demonstrate that the use of the MA-MAEC technique can yield consistent results across all the wells of the iCrystal plates. Fig. 6 shows the optical images of L-alanine crystals grown at room temperature on 21-wells of the iCrystal plates with ITO dots (a control experiment). L-alanine crystals grown on ITO dots at room temperature in 45 min reach as large as ~2 mm in size. Fig. 6 also shows that 17 of 21 wells (except well numbers: 5, 12, 16, and 17, N= the total number of L-alanine crystals grown in 21-wells = 33) resulted in the growth of L-alanine crystals of similar shape and size, which corresponds 80 ± 5% repeatability of crystallization on ITO dots at room temperature. The use of conventional microwave heating with the iCrystal plates with ITO dots resulted in 95 ± 4% (20 out 21 wells, except well number: 13, N = 37) repeatability of crystallization and the size of the L-alanine crystals were similar to those grown at room temperature, as shown in Fig. 7 & S4 (Supplementary Information). On the other hand, Fig. 8 shows that the use of focused monomode microwave heating with the iCrystal plates with ITO dots resulted in 95 ± 3% (20 out 21 wells, except well number: 3, N = 331) repeatability of crystallization. We note that the repeatability of L-alanine crystals was based on the similarity of crystal shape and size. Since the L-alanine crystals shown in Fig. 6 - 8 are placed in three-dimensional space, L-alanine crystals have different orientation with respect to the view angle of the optical microscope, and thus different crystal faces of the L-alanine crystals appear in the view. The size distribution of L-alanine crystals grown using focused microwaves was 311 ± 190 mm, which is noticeably different than those grown at room temperature (585 ± 137 mm) and conventional microwaves (542 ± 100 mm) and can be attributed to the differences in the rate of evaporation of solvent as described earlier in the text.

### Characterization of L-alanine crystals

**Fig. 9:**
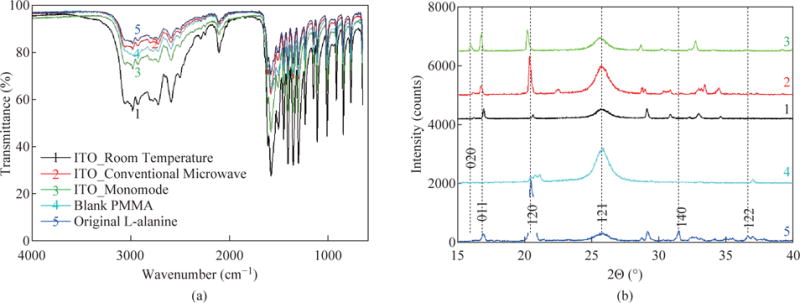
**(a)** FTIR spectra and **(b)** powder XRD patterns of L-alanine crystal grown on ITO on iCrystal plates with and without ITO (i.e., PMMA) at room temperature and after exposure to conventional microwave heating and a monomode microwave heating ITO film is on polyethylene terephthalate (PET). In addition, FTIR spectrum and XRD pattern for L-alanine powder as purchased from a vendor is given for comparison.

To demonstrate that the use of the MA-MAEC technique does not affect the chemical bonds of L-alanine during microwave heating, we have characterized L-alanine crystals using FTIR spectroscopy. FTIR spectra of the L-alanine crystals grown on ITO under different conditions, blank PMMA platform and of L-alanine powder as purchased appears to be identical as shown in Fig. 9(a). The –OH groups of L-alanine grown in all condition display a peak at 3000-2500 cm^−1^. The peaks for the –NH_2_ and –C == O groups was observed at 3500-3200 cm^−1^ and 1820-1680cm^−1^, respectively. It is also important to characterize L-alanine crystals using powder XRD analysis to determine the effect of microwave heating on the polymorphism of these crystals and/or potential conversion of the crystalline structure into an amorphous material.

Fig. 9(b) shows that the crystallinity of L-alanine remained unchanged in the presence of ITO at room temperature and after exposure to microwave heating (i.e., the MA-MAEC technique), which was assessed by the presence of identical diffraction patterns (i.e., no broadening of peaks and appearance/disappearance of additional peaks). Fig. 9(b) also shows that the diffraction patterns for L-alanine are identical on all conditions. Subsequently, these observations imply that the use of iCrystal plates with ITO dots and focused microwaves can afford scientists for the ultra-rapid crystallization of L-alanine and other amino acids in repeatable manner. Our research laboratory is currently working on the crystallization of peptides and protein on the iCrystal plates with ITO and these results will be reported in due course.

## Conclusions

The use of iCrystal plates with ITO dots (5 mm in diameter) and focused monomode microwaves at 8 GHz was demonstrated to be the most rapid method to grow L-alanine crystals in a repeatable and high throughput manner. The size of ITO films equal to the diameter of the iCrystal plates (5 cm) was found to be inefficient due to sustained damage occurred during microwave heating. The size of L-alanine crystals was observed to be depend on the type of the platform and the microwave heating method: largest size (585 ± 137 mm) at room temperature in 160 min > larger size (542 ± 100 mm) with conventional microwave oven in 55 min > smallest size (311 ± 190 mm) with focused monomode microwave in 3 min. These observations were attributed to the difference in the rate of evaporation of the solvent, where the slowest rate was observed for crystallization experiments carried out at room temperature and the fastest rate of evaporation was observed with focused monomode microwave heating. The use of focused monomode microwave heating and conventional microwave heating with the iCrystal plates with ITO dots resulted in 95 ± 3% (20 out 21 wells) and 95 ± 4% (20 out 21 wells) repeatability of crystallization, respectively. Conversely, the total number of L-alanine crystals (N) grown on ITO was increased as the rate of evaporation of the solvent was increased, N = 37 for room temperature to N = 331 for focused monomode microwave heating. Characterization of L-alanine crystals grown on ITO and blank PMMA (a control surface) with FTIR spectroscopy and XRD analysis revealed that microwave heating of L-alanine solution during crystallization on ITO did not affect L-alanine’s crystallinity, crystal structure and chemical structure. Theoretical simulations for the binding of L-alanine to metal and metal oxide surface reveal formation of no hydrogen bonds between ITO and L-alanine molecules and a binding energy of −34.9 kcal.

## Supplementary Material

Figures S1-S3
