# Supplementary material for: Ultra-Rapid Crystallization of L-alanine Using Monomode Microwaves, Indium Tin Oxide and Metal-Assisted and Microwave-Accelerated Evaporative Crystallization
Source: Nano Biomed Eng. Author manuscript; Available in PMC 2018 Apr 12. (PMC5897105; doi:10.5101/nbe.v9i2.p112-123)
Supplement: Figures S1-S3 [file NIHMS955838-supplement-Figures_S1-S3.pdf]

Supporting Information for

Ultra-Rapid Crystallization of L-Alanine Using Monomode Microwaves, Indium Tin Oxide and Metal-Assisted and Microwave-Accelerated Evaporative Crystallization

Carisse Lansiquot [1], Zainab Boone-Kukoyi [1], Raquel Shortt [1], Nishone Thompson [1], Hillary Ajifa [1], Bridgit Kioko [1], Edward Ned Constance [1], Travis Clement [1], Birol Ozturk [2], Kadir Aslan [1]*

[1] Department of Chemistry, Morgan State University, 1700 East Cold Spring Lane, Baltimore, MD 21209, USA.

[2] Department of Physics and Engineering Physics, Morgan State University, 1700 East Cold Spring Lane, Baltimore, MD 21209, USA.

* Corresponding author. E-mail: Kadir.Aslan@morgan.edu;   Tel.: +1 443 885 4257

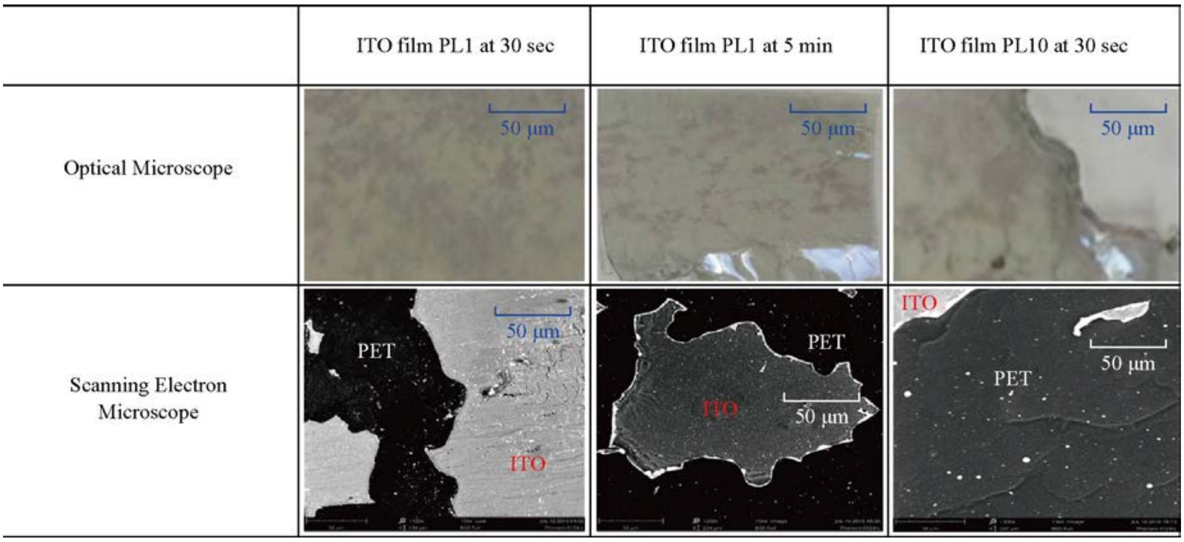

Fig. S1 Optical and SEM images of 5 cm ITO after exposure to continuous microwave heating using a conventional microwave oven at power level (PL) 1 and 10 for 30 sec and 5 min to determine the optimum microwave power level and duration for crystallization experiments without damaging ITO.

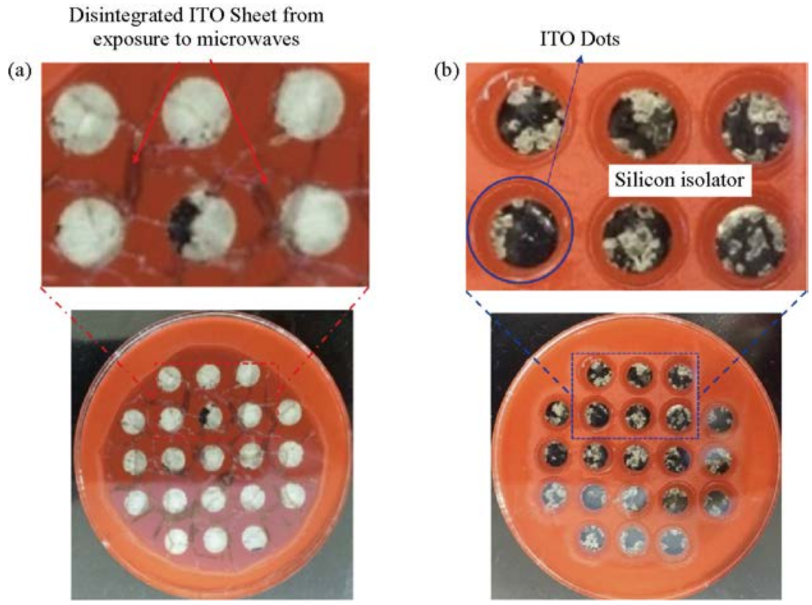

Fig. S2 Real-color photographs of **(a)** ITO film (4 cm diameter) damaged from microwave exposure due charge build up in the ITO film and **(b)** ITO dots (5 mm) with no damage from microwaves. L-alanine crystals are clearly visible on ITO dots, which indicates ITO dots can be used with the MAMEC technique.

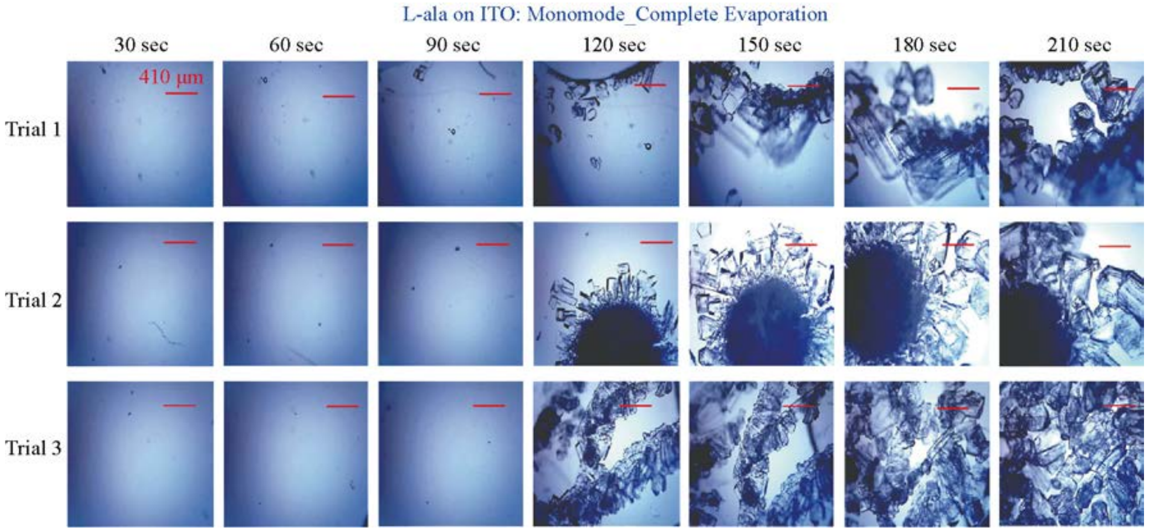

Fig. S3 Timed optical images of L-alanine crystals grown on iCrystal plates with ITO during exposure to monomode microwave heating. Each experiment in this study is repeated a minimum of three times. This figure demonstrates repeatability of the MA-MAEC technique.

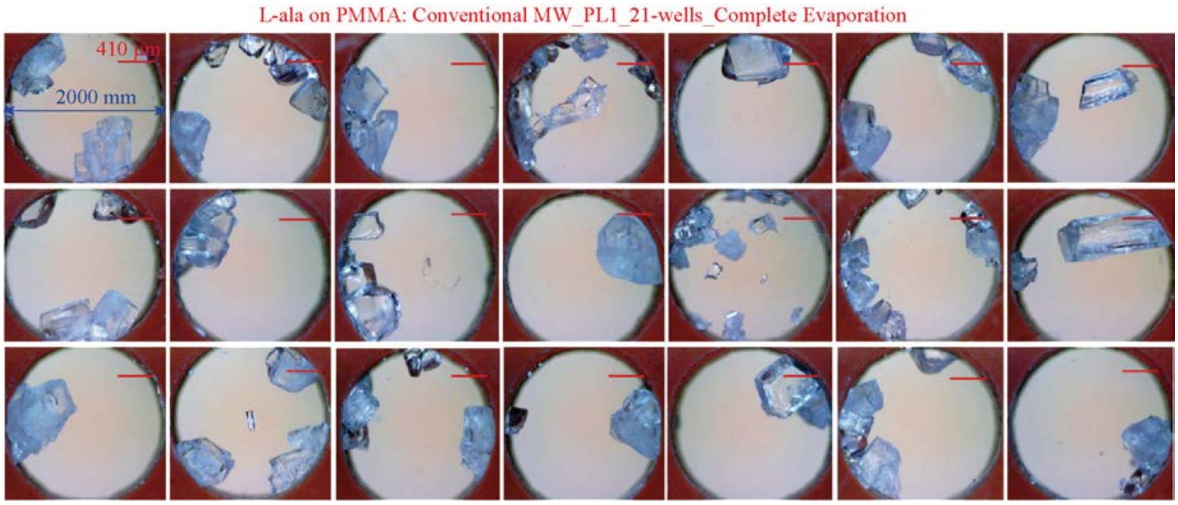

Fig. S4 Real-color images of L-alanine crystals grown on the 21 wells of the iCrystal plates with ITO using a conventional microwave oven at power level (PL) 1 after the complete evaporation of the solvent.
